# Toxicity of ivermectin to bed bugs (*Cimex hemipterus)* and risk factors associated with infestation in Kwale County, coastal Kenya

**DOI:** 10.1186/s13071-025-06836-6

**Published:** 2025-07-08

**Authors:** Tobias Odongo, Isaiah Omondi, Caroline Wanjiku, Miguel Okoko, Caroline Kiuru, Mercy Kariuki, Isaac Ringera, Bruno Otieno, Festus Mure, Joanna Furnival-Adams, Almudena Sanz Gutierrez, Rachel Otuko, Nelly Regina Rabinovich, Joseph Mwangangi, Carlos Chaccour, Marta Ferreira Maia

**Affiliations:** 1 KEMRI: Kenya Medical Research Institute, Nairobi, Kenya; 2 KEMRI-Wellcome Trust Research Programme: Centre for Geographic Medicine Research Coast, Kilifi, Kenya; 3 ISGlobal, Barcelona, Spain; 4 Facultat de Medicina I Ciències de La Salut, Universitat de Barcelona (UB), Barcelona, Spain; 5 Nuffield Department of Medicine, Centre for Tropical Medicine and Global Health, University of Oxford, Oxford, UK; 6 Navarra Center for International Development, Universidad de Navarra, Pamplona, Spain; 7 Harvard T.H. Chan School of Public Health, Boston, MA USA

**Keywords:** Ivermectin, Endectocides, *Cimex* spp., *Cimexhemipterus*., Bed bugs, Kwale, Kenya

## Abstract

**Background:**

Bed bugs (*Cimex* spp.) are obligate ectoparasites that have long been associated with human dwellings, causing discomfort and psychosocial distress. Conventional control strategies relying on insecticides are increasingly challenged by resistance, necessitating alternative interventions. Ivermectin, an endectocide known to impact various neglected tropical diseases and hematophagous arthropods, is currently being assessed for malaria vector control. This study aimed to evaluate the toxicity of ivermectin on *Cimex hemipterus*, the predominant bed bug species in Africa, within the framework of the Broad One Health Endectocide-based Malaria Intervention in Africa (BOHEMIA) project in Kwale, Kenya.

**Methods:**

A cross-sectional survey was conducted in 352 households to obtain information on self-reported bed bug infestations, socioeconomic status, and household characteristics. Bed bugs were collected from 40 infested households. After collection, bed bugs were acclimatized and exposed to blood meals spiked with ivermectin at five concentrations (85 ng/ml, 64 ng/ml, 43 ng/ml, 21 ng/ml, and 11 ng/ml), corresponding to expected serum levels 4 h to 6–7 days following a 400 µg/kg oral dose. Mortality and fecundity were monitored over a 14-day period. Statistical analyses, including Cox proportional hazard models and probit regression, were applied to assess dose–response relationships.

**Results:**

Bed bug infestation was common, with 75% of participating households reporting their presence, with infestations being strongly associated with the number of people residing in a household. Ivermectin exposure resulted in significant dose-dependent mortality in *Cimex hemipterus*, with the higher concentrations (43, 64, and 85 ng/ml) inducing over 90% mortality within 3 days postfeeding. Bed bugs that ingested blood meals containing sublethal doses of ivermectin did not lay eggs. Kaplan–Meier survival analyses demonstrated a clear inverse relationship between ivermectin concentration and bed bug survival.

**Conclusions:**

These findings provide evidence that ivermectin, administered as part of a mass drug administration campaign, could contribute to bed bug control alongside its intended impact on other diseases or vectors. The results underscore the potential for integrated public health approaches leveraging endectocide interventions. Further field evaluations in diverse locations are needed to determine the optimal number of administrations and treatment intervals required for complete infestation elimination.

**Graphical Abstract:**

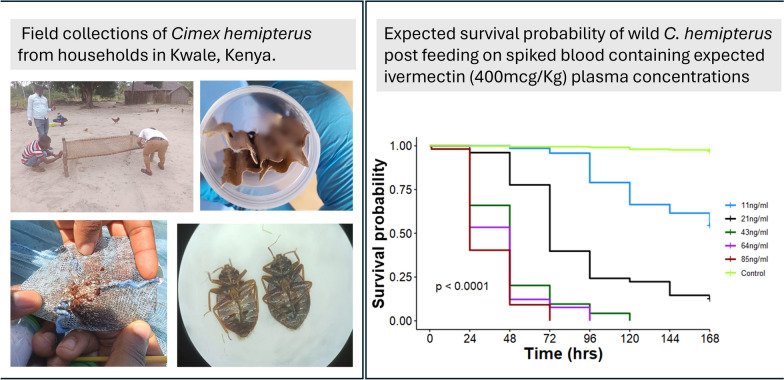

**Supplementary Information:**

The online version contains supplementary material available at 10.1186/s13071-025-06836-6.

## Background

Bed bugs (*Cimex* spp.) have infested human households since the beginning of recorded time and continue to burden human populations worldwide [1]. Bed bugs are obligate ectoparasites that feed on mammalian blood mainly during the night. They inhabit dark areas inside households, often hiding in inaccessible places such as cracks in the walls, floors, or furniture [2]. Their bites are an important source of nuisance, causing itchy skin, and rashes [3]. Scratching lesions can result in secondary bacterial infections, and potentially more severe sequelae. Beg bugs have been found to harbor various disease-causing pathogens including bacteria such as *Bartonella quintana* and methicillin-resistant *Staphylococcus aureus*, hepatitis B and C viruses, and parasites such as *Trypanosoma cruzi*. [36]. However, no role in transmission has been demonstrated to date [36]. In addition to the skin reactions and secondary infections, individuals living in infested households often experience psychosocial symptoms including anxiety, insomnia, shame, and stigmatization. Worldwide, there are over 110 species of bed bugs, however only *Cimex lecturalius* and *Cimex hemipterus* are known to feed on human blood [4]. *Cimex hemipterus* tends to occur in tropical and subtropical regions, while *C. lecturalius* is more common in temperate climates. Both male and female bed bugs need blood to molt (nymphal stages), reproduce (adults), and survive. However, they can endure prolonged periods without feeding, aided by desiccation tolerance strategies and the ability to go into quiescence [37]. While only limited data on bed bug prevalence exists, bed bugs have been found worldwide and are prevalent across most of Africa [5–8], Europe [9–11], Asia [12–16], and the Americas [17–19]. Controlling infestations can be extremely difficult and laborious in lower-middle resource settings [20]. The use of insecticide sprays has led to widespread resistance in bed bugs to virtually every insecticide class except pyrroles; whose efficacy, particularly for *C. lectularius*, is also low [22]. Furthermore, the continuous exposure to insecticides in nets used for malaria vector control in the past decades have likely increased resistance in bed bugs [21]. Within the last decade, inorganic desiccant dusts such as diatomaceous earth (DE) and silica-based dust have emerged as an attractive alternative for bed bug control [22, 23]. Desiccant dusts kill bed bugs by rapidly inducing dehydration, leading to death. Their mode of action is largely unaffected by common insecticide resistance mechanisms; however, evidence suggests that cuticle thickening, seen in insecticide-resistant bed bugs, may offer some protection by delaying mortality following dust application [24]. Notably, in moist environments, their effectiveness is reduced, as humidity impairs their ability to absorb lipids from the insect cuticle [25]. This poses a significant limitation in many African regions, which are predominantly tropical and humid—especially in households with adobe or other moisture-retaining wall materials. Additionally, the success of this approach is further hindered by the cryptic nature of bed bugs, which often seek refuge in a variety of hard-to-reach places, such as wall holes, floor cracks, and furniture crevices. Other nonchemical control methods include exposing furniture to direct sunlight, diesel, paraffins, or herbal medicines and boiling bedding with signs of infestation [5]. Some of these methods not only are ineffective but also can interfere with a household’s regular mosquito net usage and integrity, potentially leading to increased exposure to malaria [26, 27].

Endectocides are drugs that are active against endo- and ecto-parasites. Several studies have investigated different endectocide drug classes against bed bugs, including avermectins, isoxazolones, and macrocyclic lactones. Moxidectin, a macrocyclic lactone, regularly used in the veterinary field against heartworm and intestinal helminths, was evaluated in controlled conditions, demonstrating only a moderate killing effect [28]. However, it disrupted several insect physiological processes including blood digestion and egg laying up to 10 days post-blood feeding on the treated blood. A study evaluating fluralaner (Bravecto^®^), an isoxazalone, in *Cimex lectularius* control in chickens found that it caused rapid and sustained bed bug mortality up to 28 days after oral administration, suggesting its potential as a long-acting systemic treatment in poultry operations [29]. However, it is not approved for human usage. Several laboratory studies evaluated the toxicity of ivermectin-treated rodents (mice and rabbits) for bed bug control demonstrating that a significant impact on the survival and development of laboratory-reared *Cimex lectularius* post-blood feeding. These studies reported over 90% mortality rates, along with notable reductions in refeeding rates, mobility, molting, and oviposition among the bed bugs [30, 31]. One study investigated the use of ivermectin in humans for bed bug control [31]; *Cimex lectularius* was fed on blood from human subjects who had ingested a single 200 µg/kg dose of ivermectin. The results demonstrated significant reductions in bed bug population growth and fecundity for up to 96 h post-ivermectin ingestion, indicating the potential of ivermectin as a systemic agent for bed bug control in human hosts. The use of ivermectin for this purpose may be particularly attractive in areas where it is readily used in mass drug administration (MDA) campaigns against various neglected tropical diseases (NTDs) including lymphatic filariasis, onchocerciasis and soil-transmitted helminths. As a human endectocide, it is approved for the control of lice [32] and scabies [33, 34], potentially enabling a faster regulatory approval for bed bug control. Furthermore, currently, ivermectin MDA is being evaluated as a malaria control tool by targeting mosquito vectors [35–37]. Specifically, it kills Anopheline mosquitoes when they ingest a blood meal from a treated host [37–39]. Such an intervention may have a collateral impact on other hematophagous insects, including bed bugs. If ivermectin were to be effective against *C. hemipterus*, the most prevalent bed bug species in Africa, the scale-up of ivermectin MDA for malaria vector control could have significant benefits for bed bug control as part of an integrated vector control strategy.

In the context of malaria control programs, bed bug infestations have been associated with skepticism and ensuing low acceptability of malaria control measures such as indoor residual spraying (IRS) and long-lasting insecticidal nets (LLINs) [26, 40]. This is due to either (1) *Cimex* spp. attaining tolerance or resistance and resurging in the presence of insecticides still effective against mosquitoes [41–44], hence giving the impression that insecticides are not working or (2) showing increased mobility when exposed to an irritant insecticide, thus suggesting that insecticides worsen the infestation [40], or even that the nets came with bed bugs and are the cause of the infestation [45]. Public health authorities should recognize the impact of bed bug infestations on malaria control, as their presence may discourage proper net use. Infestations can lead people to avoid using nets, sleeping outdoors [46], excessively washing or boiling nets [40], or even rejecting them altogether owing to their association with bed bugs.

The present study aimed to evaluate prevalence of bed bug infestation in households of the study site (Pongwe Kikoneni and Ramisi wards) in Kwale County, Kenya as well as the toxicity of ivermectin on mortality and fecundity of wild-caught *C. hemipterus* collected in the context the Broad One Health Endectocide-based Malaria Intervention in Africa (BOHEMIA) project; a cluster randomized controlled trial evaluating ivermectin MDA for malaria control in Kwale County, Kenya [47] (trial registration: ClinicalTrials.gov NCT04966702 and PACTR202106695877303).

## Methods

### Study area

The study took place in the Pongwe-Kikoneni (4°26′36."S 39°18′27"E) and Ramisi (4°31′45.9"S 39°23′43.0"E) wards of Kwale County, Kenya. Kwale County is the most southern county on the Kenyan coast; it is located between 38.44° and 39.64°E and 3.56° and 4.70°S, bordering Tanzania to the southwest and the Indian Ocean to the east. It has a tropical climate characterized by year-round hot temperatures (25.97–32.48 ℃), high humidity averaging 82%, bimodal rainfall including long rains (March to May) and short rains (October to December), and a dry season for the remaining months. The county has a population density of 100/km^2^ [48], predominantly belonging to the Mijikenda clans as well as smaller ethnic groups, including the Kamba. Most of the population (75%) live in rural areas and are primarily dependent on subsistence farming and fishing. Interestingly, the county is also home to Diani Beach, one of Kenya’s most vibrant destinations for both domestic and international tourists. Despite its touristic and agricultural potential, it ranks among the poorest counties nationally, with 70% of residents living in houses made of earthen floor, thatched with grass and coconut palms.

### Cross-sectional study design

This study was nested within an open-label, assessor-blind, two-arm cluster-randomized trial that took place in the subcounties of Msambweni and Lungalunga in Kwale County, a coastal region in southeast Kenya. A baseline cross-sectional survey on ectoparasitic infections was conducted prior to the rolling out of the ivermectin MDA in October 2023. This included self-reported bed bug prevalence in randomly selected households participating in the BOHEMIA clinical trial, which evaluated the mass drug administration (MDA) of ivermectin for malaria control. The BOHEMIA trial was cluster randomized with two arms in which all eligible residents were asked to participate in MDA campaigns of either (1) ivermectin (400 µg/Kg) once a month for 3 months before the rainy season or (2) albendazole 400 mg (control) following the same schedule. Data on self-reported bed bug infestation were collected from 2,393 participants from 352 participating households randomly selected from 30 clusters of the BOHEMIA trial (1:1). Information on self-reported bed bug infestation, domestic animals, and their household wall type and roof type was collected. A household was considered prevalent for bed bug infestation if at least one respondent residing in that household reported the presence of bed bugs. Wealth index was ranked into four quartiles using information on asset ownership, income, and household characteristics as described by Xue et al. [49]

### Bed bug collection

**Fig. 1:**
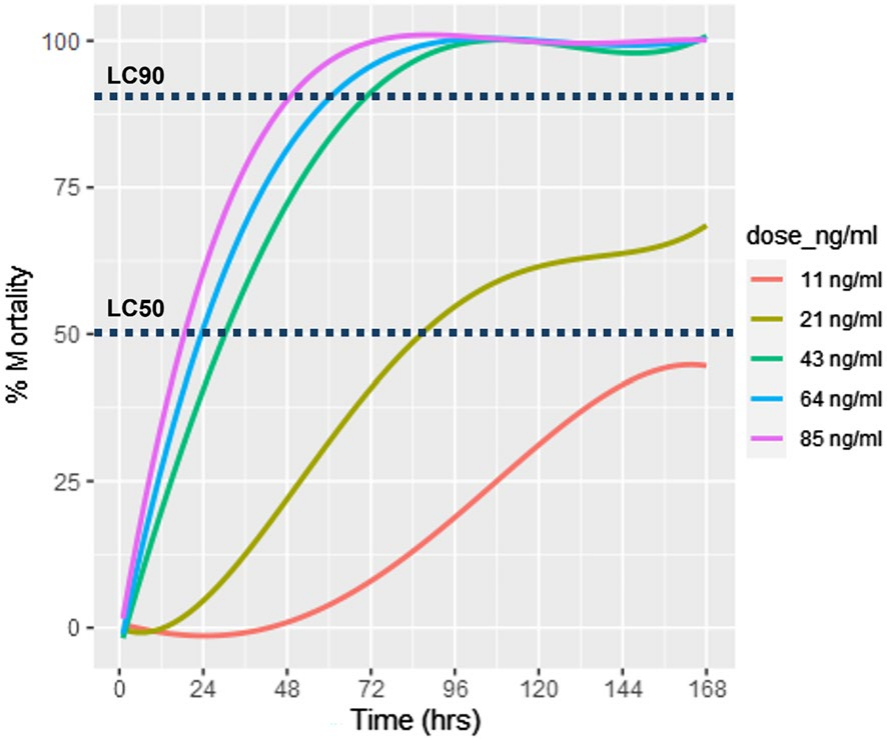
Dose–response plot of bed bugs after feeding on blood spiked with ivermectin expected plasma concentrations of ivermectin (11 ng/ml, 21 ng/ml, 43 ng/ml, 64 ng/ml, and 85 ng/ml) up to 7 days postexposure

**Fig. 2:**
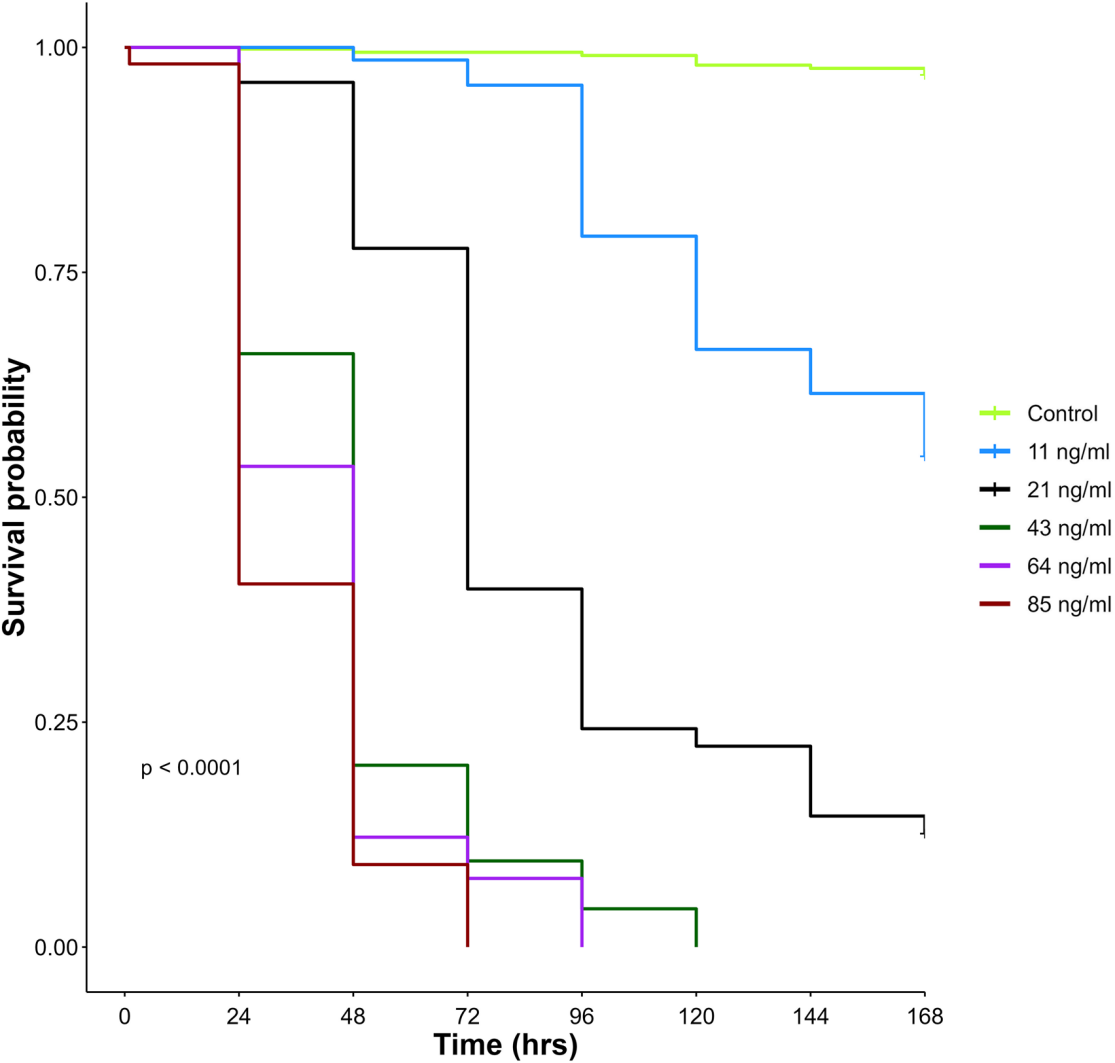
Survival curves for bed bugs as a function of the ivermectin concentrations: 0 (control), 11, 21, 43, 64, and 85 ng/ml administered via blood feeding

From mid-May to mid-June 2024, a total of 40 participating households from the control arm of the BOHEMIA trial were inspected for signs of bed bug infestations: blood stains resulting from bed bugs being crushed, black spots indicative of excrement, miniscule pale-yellow eggs or their casings, and live bed bugs. Typical bed bug resting sites were carefully examined for purpose of bed bug collection using brush sweeping method, including beds, walls, mattresses, furniture, and nets (Figs. 1 and 2 in Electronic Supplementary Material). Moveable materials were taken outside for sufficient light during inspection. When seen, live bed bugs were swept into a 120 ml plastic collection cup using a camel hairbrush (Fig. 2 in the Electronic Supplementary Material). A maximum of 50 bed bugs were collected in each container, provided with folded piece of dry cardboard paper for refuge, and the containers were secured using a finely perforated lid for aeration. Collection cups were then transferred to a cooler box at ambient temperature and transported to a field insectary adapted for this experiment. The insectary was kept at local indoor ambient temperatures, with these ranging at the time of the experiment between 24 and 29 °C.

### Bed bug identification

Bed bugs were morphologically identified under a stereomicroscope (Nikon C-SP, Japan) according to the Usinger 1996 identification keys [50]. Bed bugs were identified to sex and species, with females having a rounded abdominal tip and males having a pointed abdominal tip. The main external differences between tropical bed bugs (*C. hemipterus*) and common bed bugs (*C. lectularius*) consist in the relative width of the pronotum (in *C. hemipterus*, it is less than 2.5 times wider than its length measured along the midline; in *C. lectularius*, it is more than 2.5 times wider than its length) and the width of the extended and flattened lateral margins of the pronotum or paranotum (in *C. hemipterus*, they are much narrower than in *C. lectularius*).

### Bed bug selection for ivermectin efficacy assays

In the insectary, the bed bugs were left unfed for 7 days to digest any blood that might have been picked from the field and to acclimatize. A total of 50 unfed live adults (male and females of unknown age) were selected and placed into new plastic cup with a straight hard paper inserted in the middle to help bed bugs reach the top for blood meal and covered with a fine net held in place by rubber band. Each container was labeled with the concentration of ivermectin to be administered or control. Remaining immature and blood-fed bed bugs were killed by immersing them in boiling water.

### Sample size calculation for IVM toxicity

Considering time to event outcomes based on an exponential survival model, we used the Schoenfeld formula below to determine the sample size for the exposed and unexposed assays [51].

$$\it \it {\text{n = }}\,\left( {{1 \mathord{\left/ {\vphantom {1 {{\text{p}}_{{\text{o}}} \, + \,{1 \mathord{\left/ {\vphantom {1 {{\text{p}}_{{1}} }}} \right. \kern-0pt} {{\text{p}}_{{1}} }}}}} \right. \kern-0pt} {{\text{p}}_{{\text{o}}} \, + \,{1 \mathord{\left/ {\vphantom {1 {{\text{p}}_{{1}} }}} \right. \kern-0pt} {{\text{p}}_{{1}} }}}}} \right) * \,\left( {\left( {{{{\text{Z}}_{{{1 - }\alpha {/2}}} {\text{ + Z}}_{{{1 - }\beta }} } \mathord{\left/ {\vphantom {{{\text{Z}}_{{{1 - }\alpha {/2}}} {\text{ + Z}}_{{{1 - }\beta }} } {{\text{logHR}}}}} \right. \kern-0pt} {{\text{logHR}}}}} \right)^{ \wedge \,2} } \right)\,$$

The following sample size considerations were used:

- The probability of mortality for a bedbug in unexposed assay, *P*_o_ = 10%.
- The probability of mortality of a bedbug in exposed assay, *P*_1_ = 22%.
- The hazard ratio of three for bed bugs in exposed assay compared with an unexposed assay.
- A 5% significance level.
- An intention to achieve 80% statistical power: *Z*_1 − *α* / 2_ = 1.96 and *Z*_1 − *β*_ = 0.84.

Bed bugs were assigned to either the control (unexposed) or exposed groups in a 1:1 ratio, with approximately 500 bed bugs in the unexposed group and the remaining 500 distributed evenly across the different concentrations in the exposed assays. We randomly allocated approximately 100 bed bugs per each of the five different exposure assays, resulting in final group sizes of 143 for the 11 ng/mL assay, 103 for the 21 ng/mL assay, 94 for the 43 ng/mL assay, 131 for the 64 ng/mL assay, and 109 for the 85 ng/mL assay. Each assay was completed once the target sample size of approximately 100 bed bugs was reached per group within 6–8 replicates. A balanced contemporaneous control was used for each replicate.

### Ivermectin concentrations and spiked-blood serial dilutions

Toxicity was tested across five ivermectin concentrations—11 ng/mL, 21 ng/mL, 43 ng/mL, 64 ng/mL, and 85 ng/mL—corresponding to the expected serum levels at C_12.5_, C₂₅, C₅₀, C₇₅, and Cₘₐₓ, respectively, following a single oral dose of 400 µg/kg, as administered in the BOHEMIA clinical trial. These concentrations were informed by a prior pharmacokinetic study evaluating 400 µg/kg of ivermectin in Kenyan adults [39]. The range of concentration tested from 85 to 11 ng/mL reflects expected blood levels approximately 4 h (Cₘₐₓ—85 ng/ml) to 6–7 days (C_12.5_—11 ng/ml) postingestion of 400 µg/kg of ivermectin. A stock solution containing 1,000 ng/ml ivermectin was prepared by pipetting 5 μl of ivermectin injection 1%^®^ (Osho Chemical Industries, Kenya) into a Falcon tube, topped-up with distilled water to 50 ml, and mixed by vigorous shaking. In total, 12 ml of blood was obtained from healthy donors (18–40 years) and preserved in heparin tubes. A total of six individual 15-ml sterile Falcon tubes were labeled with the individual ivermectin concentrations and date of experiment. Thereafter, the respective volumes of the ivermectin stock solution (55 µl, 105 µl, 215 µl, 320 µl, and 425 µl) were dispensed into the labeled Falcon tubes and topped up with the heparinized donor blood to 5 ml to obtain the desired ivermectin concentrations and thoroughly mixed. The control tube contained 5 ml of same untreated blood. All dilutions were done at room temperature.

### Membrane feeding assay using ivermectin-spiked blood

An inverted cup feeding method was used to administer the blood to the bed bugs. A disposable 50 ml paper cup with a diameter of 2.5 cm and a depression (~0.1 cm) at the bottom was used. A total of 2 ml of ivermectin-spiked blood was dispensed into the bottom wedge of the paper cup and covered with a stretched plumber’s tape as a membrane. The cup was then held in an upright position and placed on top of the bed bug-holding cup ensuring that the membrane was in contact with the netting covering the mouth of the holding cup. The sensor of a thermometer (HTC-2) was placed in the plastic cups and warm water added to bring the blood to a temperature of 37–40 °C. The membrane and bed bug-holding cups were then carefully placed in a closed cooler box to provide a dark and cool environment and allowed to feed. After 2 h, all bed bugs were released in a cage and engorged bed bugs separated from unfed individuals and put in a clean holding cup. All unfed bed bugs were killed with no further investigation.

### Survival and oviposition monitoring

Survival monitoring began 1 h post-blood feeding and continued for seven consecutive days, which is the period required for a bed bug to digest a blood meal and re-feed (Table 1). Bed bugs unable to walk or those that fell over their back 1 h postfeeding were recorded as having been knocked-down, and bed bugs that were stiff on their back and legs folded over their abdomen with no moving body parts were recorded as dead. Daily mortality was measured throughout the monitoring period. All dead bed bugs were placed in a 2-ml microtube with a silica gel, labeled with ivermectin concentration, date, and either dead or alive and stored for further analysis. The live bed bugs were monitored for oviposition by checking for the presence of eggs 24 h postfeeding to up to 14 days. The bed bugs were kept in the holding cups at room temperature during the monitoring period. Oviposition monitoring was stopped when either all bed bugs were dead or oviposition was observed. After the 14th day, all live bed bugs were killed by freezing at −20 °C for 24 h and stored in microtubes containing desiccant for further analysis.

**Table 1:** Descriptive statistics of the study households and their bivariate association to self-reported bed bug prevalence

Variable	No bed bugs (*N* = 88)	Bed bugs (*N* = 264)	Total (*N* = 352)	*χ*^2^	*P*-Value
Cattle present					
No	62 (24.2%)	194 (75.8%)	256 (72.7%)		
Yes	26 (27.1%)	70 (72.9%)	96 (27.3%)	0.172	0.678
Number of cattle					
Mean (standard deviation)	5.46 (3.91)	6.75 (7.06)	–		0.889
Median (min–max)	4.50 (1.00–19.0)	4.00 (1.00–41.0)			
Household size					
Mean (standard deviation)	5.08 (2.32)	6.01 (3.03)	–		0.017*
Median (min–max)	5.00 (1.00–11.0)	6.00 (1.00–17.0)			
Cattle overnight < 50 m					
No	12 (30.8%)	27 (69.2%)	39 (40.6%)		
Yes	14 (24.6%)	43 (75.4%)	57 (59.4%)	0.192	0.661
Wealth index					
High	43 (35.2%)	79 (64.8%)	122 (34.7%)		
Medium	17 (21.5%)	62 (78.5%)	79 (22.4%)	10.669	0.005*
Low–poorest	28 (18.5%)	123 (81.5%)	151 (42.9%)		
Roof type					
Metal sheets	53 (31.2%)	117 (68.8%)	170 (48.3%)		
Palm or grass thatch	35 (19.2%)	147 (80.8%)	182 (51.7%)	6.068	0.014*
Wall type					
Blocks and cement	38 (30.2%)	88 (69.8%)	126 (35.8%)		
Mud	50 (22.1%)	176 (77.9%)	226 (64.2%)	2.373	0.123

^*^Statistically significant

### Statistical analysis

Data gathered during the household level cross-sectional survey was collected using Open Data Kit (ODK) and later exported to .csv format (Microsoft Windows Excel 2016). The data resulting from bed bug blood-feeding assays was collected on data collection forms and then entered electronically entered onto a .csv file. All data was exported to R version 4.4.1 (R Foundation for Statistical Computing, Vienna, Austria) for analysis.

Categorical variables were presented as frequencies and percentages, while continuous variables were presented as means and standard deviations. Principal component analysis (PCA) was applied to ownership of assets at the household level to generate the wealth index/socioeconomic status (SES) as described by Xie et al. [49]. A chi-squared test was used for comparisons between household characteristics and self-reported bed bug prevalence at 0.05 significant levels. The outcome variable (bed bug presence) implies that at least one member of the household reported the presence of bed bugs whenever they were sleeping. A generalized linear model of binomial family and a complementary log–log link function were used to evaluate the independent association between each of the factors and self-reported bed bug prevalence (univariable model). Backward elimination was used to identify the best multivariable model. The results were presented in terms of relative risks (RR) with 95% confidence levels (95% CI).

The Cox proportional hazards model was used to analyze bed bug survival following blood feeding on ivermectin-spiked blood compared with the control. Probit regression was applied to estimate the lethal concentration needed at each time point post-blood feeding to kill 50% and 90% of the bed bugs in 7 days (LC_50_ and LC_90_ values). Survival data were visualized using Kaplan–Meier curves, while dose–response relationships were illustrated with dose–response plots. The results were reported as hazard ratios (HR) with corresponding 95% CI.

## Results

### Bed bug prevalence and associated risk factors

A total of 2,393 participants from 352 households were surveyed for self-reported presence/absence of bed bugs, and the results indicated that 264 households (75.0%) reported having bed bugs—defined as at least one resident reporting presence of bed bugs in the household. Of all households, 96 (27.3%) had cattle, of which 57 (59.4%) had the cattle sleeping within a 50-m radius from the house. On average, each household had seven cattle (median: 4, range: 1–41). Cattle ownership and cattle distance from the household were not associated with bed bug prevalence as per the chi-squared test results; 72.9% and 75.8% of the households with and without cattle, respectively, had reported having bed bugs (*χ*^2^ = *0.172, P = 0.678*), while 75.4% of the households where cattle slept within a 50-m radius compared with 69.2% of the households where cattle did not sleep within a 50-m radius of the house had bed bugs (*χ*^2^ = *0.192, P = 0.661*). Moreover, there was no significant difference in the number of cattle between the households with bed bugs and those without (*P* = 0.889—by Wilcoxon rank sum test). However, households that reported having bed bugs had significantly more members compared with those that reported not having bed bugs (*P* = 0.017—by Wilcoxon rank sum test).

The houses were primarily made of mud walls (64.2%) and palm/grass roofs (51.7%), and most of the households (42.9%) could be classified as belonging to the low SES class. Bed bug presence was reported more commonly in low SES households (81.5%) compared with high SES households (64.8%) (*χ*^2^ = *10.669, P = 0.005*). Additionally, 80.8% of the houses with palm/grass roofs reportedly had bed bugs, compared with 68.8% of the houses that had iron roofs (*χ*^2^ = *6.068, P = 0.014*), while 77.9% of the mud-walled houses compared with 69.8% of the block-and-cement-walled houses reported having bed bugs, although these differences were not significant (*χ*^2^ = 2.373, *P* = 0.123; Table 1).

In the univariable model, a unit increase in the number of members of a household was associated with a 1.07 times increased risk of having bed bugs (RR = 1.07, *P* = 0.004). The number of cattle owned, whether cattle slept within 50 m radius from the house, and the wall type of the house were all not associated with the presence of bed bugs. With respect to the wealth index, medium-class households were associated with a 1.47 times higher risk of having bed bugs (RR = 1.47, *P* = 0.034), while low-class households were associated with a 1.62 times higher risk of having bed bugs compared with higher-class households (RR = 1.62, *P* = 0.002). Palm/grass roofs were associated with a 41% increased risk of bed bug infestation compared with iron roofs (RR = 1.41, *P* = 0.010).

The final multivariable model had the number of cattle, wealth status, whether cattle slept within a 50-m radius of the house, and household occupancy size (number of residents) as possible predictors of bed bug prevalence. Roof type and wall type were excluded as they introduced multicollinearity in the model, having been used in the PCA model to create the household wealth index. Bed bug prevalence was identified to be high in households with more people (adjusted (adj.) RR = 1.22, *P* = 0.026). We, however, found no significant association between the presence of bed bugs and the number of cattle owned, household wealth index, or whether cattle slept within 50 m from the house. (Table 2).

**Table 2:** Univariable and multivariable logistic regression model with complementary log–log link function results evaluating factors associated with self-reported prevalence of bed bugs in households of Kwale, Kenya

	Univariable models		Multivariable model	
Predictors	RR (95% CI)	*P*-value	RR (95% CI)	*P*-value
Number of cattle	1.02 (0.98–1.07)	0.280	1.06 (0.97–1.20)	0.247
Cattle sleep within 50 m from house				
No	Ref		Ref	
Yes	1.19 (0.72–2.01)	0.505	1.30 (0.49–3.41)	0.599
Wealth index				
High	Ref		Ref	
Medium	1.47 (1.03–2.11)	0.034*	1.74 (0.57–5.65)	0.338
Low–poorest	1.62 (1.19–2.20)	0.002*	2.50 (0.76–9.28)	0.144
Household size (occupancy)	1.07 (1.02–1.13)	0.004*	1.22 (1.03–1.47)	0.026*
Roof type				
Iron	Ref		–	
Palm/grass	1.41 (1.09–1.84)	0.010*	–	
Wall type				
Block and cement	Ref		–	
Mud	1.26 (0.96–1.66)	0.101	–	

^*^Statistically significant

*RR* risk ratio, *95% CI* 95% confidence intervals

### Effect of ivermectin on bed bug survival

A total of 3300 unfed bed bugs were used in this study. All bed bugs were morphologically identified as *Cimex hemipterus* (Fig. 3 in the Electronic Supplementary Material). Each assay replicate was conducted with approximately 50 bed bugs (Table 3). We conducted 6–8 replicates per ivermectin group, each with a contemporaneous control. A total of 1140 (35%) successfully engorged on blood, of which 580 were fed on ivermectin-spiked blood, and 560 were fed on untreated blood (control). The remaining 2160 bed bugs did not feed and were discarded from the experiment. A total of 143, 103, 94, 131, and 109 were exposed to 11 ng/ml, 21 ng/ml, 43 ng/ml, 64 ng/ml, and 85 ng/ml concentrations of ivermectin respectively. Daily mortality and the corresponding values for LC_50_ and LC_90_ are presented in Table 4 and Fig. 1. Bed bug survival was high in the control group, with the majority surviving throughout the seven experimental days. When exposed to a plasma concentration above 67 ng/ml, 50% of bed bugs died within 24 h and 90% within 48 h. At day 7, the hazard ratio (HR) for bed bug mortality in the ivermectin groups compared with the control group ranged between 18.7 for the 11 ng/ml and 530 for the 85 ng/ml group (Table 3 and Fig. 2). More than 90% of the bed bugs that fed on 43, 64, or 85 ng/ml died within 3 days. At 7 days post-blood feeding, some of the bed bugs that had fed on lower concentrations (11 and 21 ng/ml) were still alive, with around 12.6% survival in the 21 ng/ml group and 54.5% in the 11 ng/ml group.

**Table 3:** Number of replicates and total number of bed bugs used in the ivermectin toxicity assays showing results from Cox regression model assessing the effect of different ivermectin concentrations on bed bug survival

Concentration^1^	Replicates	*n*	HR	95% CI	*P*-value
Control	33	559	Ref	–	–
11 ng/ml	6	143	18.7	10.9, 31.9	< 0.001*
21 ng/ml	7	103	76.7	45.3, 130	< 0.001*
43 ng/ml	6	94	277	161, 478	< 0.001*
64 ng/ml	8	131	374	218, 643	< 0.001*
85 ng/ml	6	109	530	305, 923	< 0.001*

^*^Statistically significant

^1^The range of concentration tested from 85 to 11 ng/mL reflects expected blood levels approximately 4 h (Cₘₐₓ—85 ng/ml) to 6–7 days (C_12.5_—11 ng/ml) postingestion of 400 µg/kg of ivermectin [39]

n total number of bed bugs, *HR* hazard ratio, *95% CI* 95% confidence interval

**Table 4:** Probit regression results: LC_50_ and LC_90_ at various time intervals postexposure

Time post exposure	LC_50_	LC_90_
1 h	96.0 ng/ml^1^	103.5 ng/ml^1^
Day 1	67.2 ng/ml	166.1 ng/ml^1^
Day 2	32.5 ng/ml	67.9 ng/ml
Day 3	24.9 ng/ml	48.6 ng/ml
Day 4	17.7 ng/ml	34.8 ng/ml
Day 5	15.2 ng/ml	31.5 ng/ml
Day 6	13.9 ng/ml	39.3 ng/ml
Day 7	12.7 ng/ml	28.5 ng/ml

^1^Values above 85 ng/ml are not likely to be reached in human plasma post-oral treatment with 400 µg/kg of ivermectin; however, they are presented as part of the probit model results

### Effect of ivermectin on bed bug oviposition

Bed bugs that fed on ivermectin concentrations ≥ 43 ng/ml all died within 4 days post-blood feeding, and no oviposition was observed. Bed bugs fed on 11 and 21 ng/ml of ivermectin survived for 14 and 10 days, respectively; however, no oviposition was observed. By contrast, all respective contemporaneous controls observed oviposition by day 4 (Table 5).

**Table 5:** Effect of ivermectin treatment on bed bug oviposition

Treatment	Number of female bed bugs	Monitoring time (days)	Oviposition observed	Time of oviposition
11 ng/ml	143	14	No	No eggs laid
Control	139	4	Yes	Eggs laid on day 4
21 ng/ml	103	10	No	No eggs laid
Control	115	10	Yes	Eggs laid on day 4
43 ng/ml	94	4	No	No eggs laid
Control	81	4	Yes	Eggs laid on day 4
64 ng/ml	131	4	No	No eggs laid
Control	129	4	Yes	Eggs laid on day 4
85 ng/ml	109	4	No	No eggs laid
Control	96	4	Yes	Eggs laid on day 4

Observed time to oviposition post-blood feeding on ivermectin-spiked blood compared with contemporaneous control (untreated blood)

## Discussion

Our study provides compelling evidence that ivermectin has a significant toxic effect on *Cimex hemipterus*, the predominant bed bug species in Africa. The observed dose-dependent mortality suggests that ivermectin exposure through blood meals can be a viable intervention against bed bug infestations. Our study tested a range of ivermectin blood concentrations expected following treatment with single dose ivermectin of 400 µg/kg, representing the expected decline over time due to metabolic clearance [39]. According to a previously conducted pharmacokinetic study [39], we expect ivermectin blood concentrations to range between 85 ng/ml (C_max_—expected 4 h posttreatment) and 11 ng/mL (C_12.5_ expected 6–7 days posttreatment). In the field, bed bugs will be exposed to a range of ivermectin levels—from high concentrations immediately following dosing to much lower levels several days later. Notably, our findings show that even the lowest concentration tested (11 ng/mL) was highly toxic, with a hazard ratio of 18.7 (95% CI 10.9–31.9; *P* < 0.001), resulting in nearly 50% mortality of bed bugs within 7 days postfeeding.

These findings align with previous research demonstrating the efficacy of ivermectin against other hematophagous arthropods, reinforcing its potential as a broad-spectrum endectocide. The high mortality observed within 7 days postfeeding highlights ivermectin’s rapid action on bed bugs. Moreover, bed bugs that had fed on sublethal concentrations of ivermectin-treated blood did not lay eggs, indicating that the drug also impairs bed bug reproduction, potentially contributing to long-term population suppression. These findings suggest that ivermectin’s impact extends beyond immediate mortality, influencing bed bug population dynamics over time. The results have important implications for integrating public health programs targeting NTDs that utilize ivermectin MDA with bed bug control efforts. Furthermore, a photovoice study conducted at the same site within the context of the BOHEMIA trial documented community experiences and perceptions related to the intervention. Participants randomized to the ivermectin MDA arm reported a perceived reduction in bed bug infestations, evidenced by several individuals selecting and discussing photographs of beds and dead bed bugs as representations of their experiences during the MDA [52]. These community-reported observations align with our current findings, which provide empirical evidence supporting the toxic effect of ivermectin on bed bugs. As ivermectin is currently being evaluated for malaria vector control, our study suggests that its deployment through MDA could offer additional benefits by simultaneously targeting bed bugs. This is particularly important, as bed bug infestations can impact net usage, a crucial component of malaria prevention. This dual impact could be particularly beneficial in regions where NTDs, malaria, and bed bug infestations cooccur, enhancing public acceptability and resource utilization for multiple public health benefits. In addition, they highlight the need to continuously monitor bed bug susceptibility in areas targeted with ivermectin MDA to apply the lessons learned on the role of bed bugs in the community acceptance of public health campaigns.

Houses with a higher number of residents were more likely to harbor bed bugs. These findings are in line with available evidence associating crowded living conditions with increased bed bug infestations, as higher occupancy facilitates their spread and sustains infestations over time. The potential for ivermectin as a bed bug control measure is particularly relevant for institutional settings such as boarding schools, prisons, and refugee shelters, where infestations can spread rapidly owing to high population density and frequent human contact. These environments often face challenges in controlling bed bugs using conventional strategies owing to resource and logistical constraints. Administering ivermectin through MDA in such settings could provide a scalable intervention that can be integrated with other strategies, thereby reducing bed bug infestations and their associated health and psychological impacts.

Despite these promising findings, several questions remain regarding the operational feasibility of ivermectin-based bed bug control. Further research is needed to determine the ideal dosing regimens and the sustainability of such interventions in institutional settings and the optimal number of administrations and treatment intervals necessary to achieve complete infestation elimination. Additional research is also needed to adequately measure the nonlethal effect of ivermectin and its impact on the life cycle of *C. hemipterus* to better design deployment strategies. Additionally, potential resistance development in bed bug populations should be monitored to ensure sustained efficacy.

This study has several limitations. The BOHEMIA cross-sectional study relied on self-reported household infestations with bed bugs, which may have been associated with recall bias and may have led to underestimation owing to the stigma associated with bed bug presence. Toxicity assays were conducted using blood spiked with known ivermectin concentrations representing the expected blood concentrations up to 7 days posttreatment with a single dose of ivermectin (400 µg/kg). While this approach ensures standardized exposure, it does not account for the effects of ivermectin metabolization that occur in vivo. Survival was monitored for only 7 days post-blood feeding. Although most bed bugs had died within this period, extending the observation time might have revealed prolonged effects, as observed in mosquitoes [37]. Additionally, we did not quantify refeeding rates, which ivermectin has been shown to affect in other bed bug species [30].

## Conclusions

Our study highlights the potential role of ivermectin in bed bug control, presenting a novel application for this endectocide beyond its traditional use. Integrating ivermectin-based interventions into broader public health strategies could enhance efforts to address NTDs, malaria, and bed bug infestations concurrently, ultimately improving health and well-being in affected communities.

## Supplementary Information

Supplementary material 1.

## Data Availability

The datasets generated used in the current study are available in the Harvard Dataverse repository (doi.org/10.7910/DVN/E5VVGM).
