# Supplementary material for: Toxicity of ivermectin to bed bugs (Cimex hemipterus) and risk factors associated with infestation in Kwale County, coastal Kenya
Source: Parasit Vectors. 2025 Jul 8;18:269. doi: 10.1186/s13071-025-06836-6 (PMC12239333; doi:10.1186/s13071-025-06836-6)
Supplement: Supplementary file 1 — Supplementary material 1. [file 13071_2025_6836_MOESM1_ESM.docx]

**Supplementary materials**

*Odongo et al.*

*Toxicity of ivermectin to bed bugs (*Cimex hemipterus*) and risk factors associated with infestation in Kwale county, coastal Kenya.*


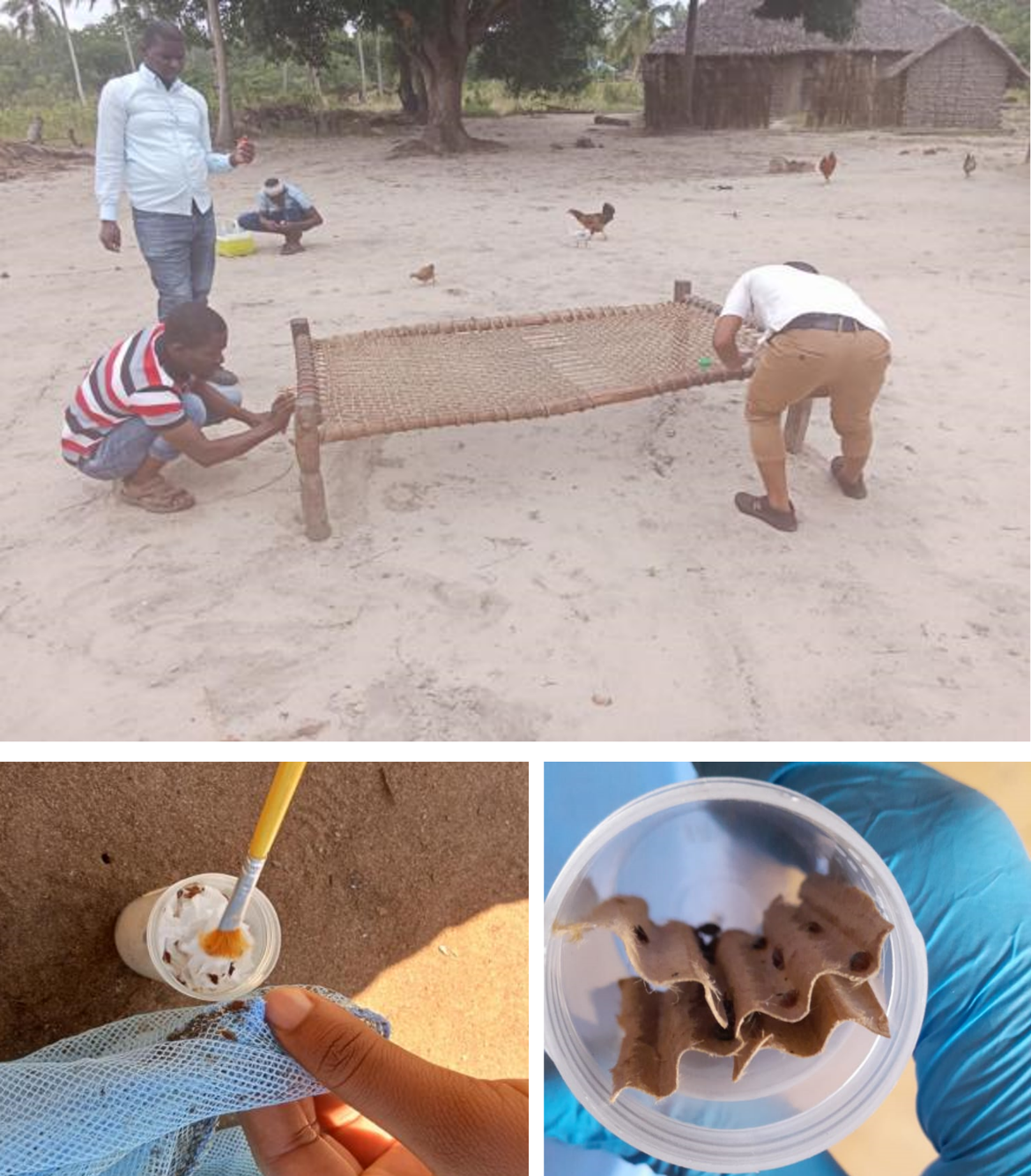


**Fig. 1** Field workers collecting bed bugs in Pongwe-Kikoneni and Ramisi wards of Kwale county, Kenya.


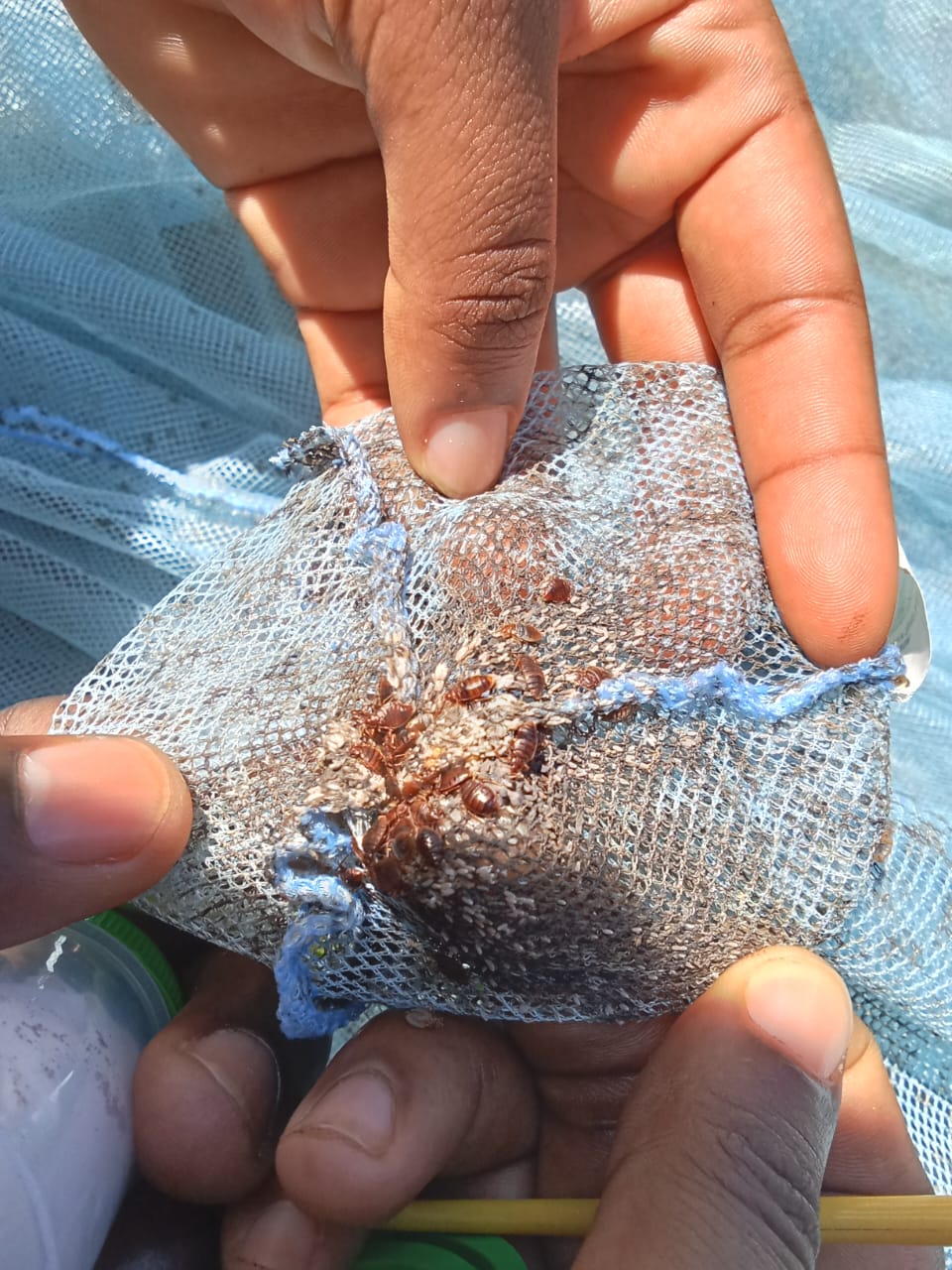


**Fig. 2** Infested net from one of the households where bed bugs were collected in the Kwale County, Kenya.


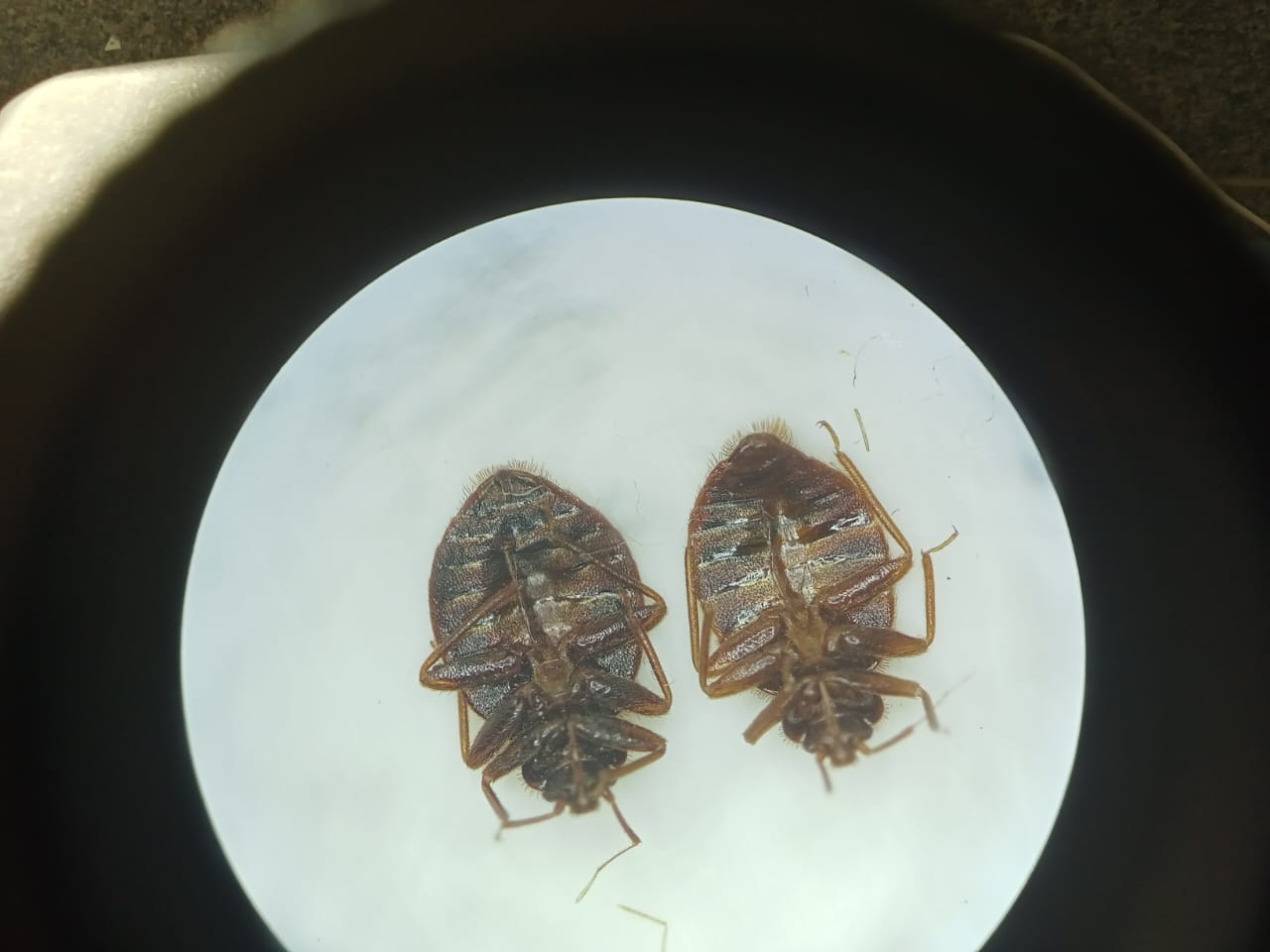


**Fig. 3** Ventral views of *Cimex hemipterus* female (right) and male (left) specimens.
